# Eutrophication Assessment Based on the Cloud Matter Element Model

**DOI:** 10.3390/ijerph17010334

**Published:** 2020-01-03

**Authors:** Yumin Wang, Xian’e Zhang, Yifeng Wu

**Affiliations:** 1 School of Energy and Environment, Southeast University, Nanjing 210096, China; 101011433@seu.edu.cn; 2 School of Environment and Municipal Engineering, North China University of Water Resources and Electric Power, Zhengzhou 450046, China; zhangxiane@ncwu.edu.cn

**Keywords:** cloud matter element (CME), eutrophication evaluation, lakes, reservoirs

## Abstract

Eutrophication has become one of the most serious problems threatening the lakes/reservoirs in China over 50 years. Evaluation of eutrophication is a multi-criteria decision-making process with uncertainties. In this study, a cloud matter element (CME) model was developed in order to evaluate eutrophication level objectively and scientifically, which incorporated the randomness and fuzziness of eutrophication evaluation process. The elements belonging to each eutrophication level in the CME model were determined by means of certainty degrees through repeated simulations of cloud model with reasonable parameters of expectation *E_x_*, entropy *E_n_*, and hyper-entropy *H_e_*. The weights of evaluation indicators were decided by a combination of entropy technology and analytic hierarchy process method. The neartudes of water samples to each eutrophication level of lakes/reservoirs in the CME model were generated and the eutrophication levels were determined by maximum neartude principal. The proposed CME model was applied to evaluate eutrophication levels of 24 typical lakes/reservoirs in China. The results of the CME model were compared with those of comprehensive index method, matter element model, fuzzy matter element model, and cloud model. Most of the results obtained by the CME model were consistent with the results obtained by other methods, which proved the CME model is an effective tool to evaluate eutrophication.

## 1. Introduction

Rapid population growth and economic rise in past decades contribute to the pollution of water bodies in China, including lakes, reservoirs, rivers, and estuaries, which leads to the deterioration of water environment. Eutrophication is a natural process in which phosphorus and nitrogen stimulate primary production, which leads to enhanced algal growth and phytoplankton blooms. Lakes/reservoir eutrophication has become one of the most serious environmental issues worldwide, especially in developing countries [1]. Assessing eutrophication level scientifically and reasonably is essential for environmental management agencies.

In the past literatures, many methods have been applied to evaluate eutrophication. However, subjectivity exists in indicators selection and weights determination in traditional eutrophication assessment. Therefore, it is reasonable and essential to interpolate multiple methods to assess eutrophication, such as principle component analysis method [2], fuzzy comprehensive assessment method [3], matter element (ME) method [4,5,6], projection pursuit method [7], artificial neutral network technology [8], support vector machine approach [9], random forest model [10], etc. These methods made eutrophication evaluation simple by using mathematic tools. Since the eutrophication assessment is a multi-criteria contradictory decision-making process, for example, some indicators belong to level A while the other indicators belong to level B, it is usually difficult to perform. The ME theory is suitable for solving such problems and can obtain better results [11]. Therefore, in this study, ME model is adopted for assessing eutrophication statuses of lakes in China [12]. In addition, during the process of eutrophication evaluation, fuzziness and randomness exist in monitor of data, selection of statistical methods, and determination of weights [13]. To solve the fuzziness problem, fuzzy mathematics theory was introduced into ME method in terms of membership functions to construct fuzzy matter element (FME) model with consideration of fuzziness and uncertainty of the eutrophication evaluation [14,15,16,17]. Unfortunately, precise membership functions in FME model is unable to give consideration to both fuzziness and randomness of objects and leads to inaccurate evaluation results since the character of fuzzy objects was described as “Both A and B” [18]. Under such circumstance, the cloud model was introduced into FME model by extending the accurate membership functions to random membership functions in terms of statistical distributions of membership functions, which is a new cognition model of uncertainty based on probability theory and fuzzy set theory [19,20,21]. The membership functions in FME model were replaced by a certain degree of cloud drops. Therefore, the cloud matter element (CME) method was generated with consideration of randomness and fuzziness of eutrophication evaluation systematically by transforming between qualitative notion and quantitative instances. The CME model takes the advantages of both cloud model and matter element model, and can be applied to assess eutrophication status, which is contradictory, uncertain, fuzzy, and random [13].

In this study, a cloud matter element (CME) model is developed in Section 2 with the combination of ME model and cloud model. The CME-based eutrophication assessment process is discussed in Section 2 including framework, determination of parameters, weights of indicators, calculation of neartude, and determination of eutrophication level. In Section 3, the proposed CME model is applied to eutrophication evaluation of 24 typical lakes/reservoirs of China, and the results of CME model were compared with the other methods. In Section 4, conclusions were stated.

## 2. Methodology

### 2.1. Study Area

**Figure 1:**
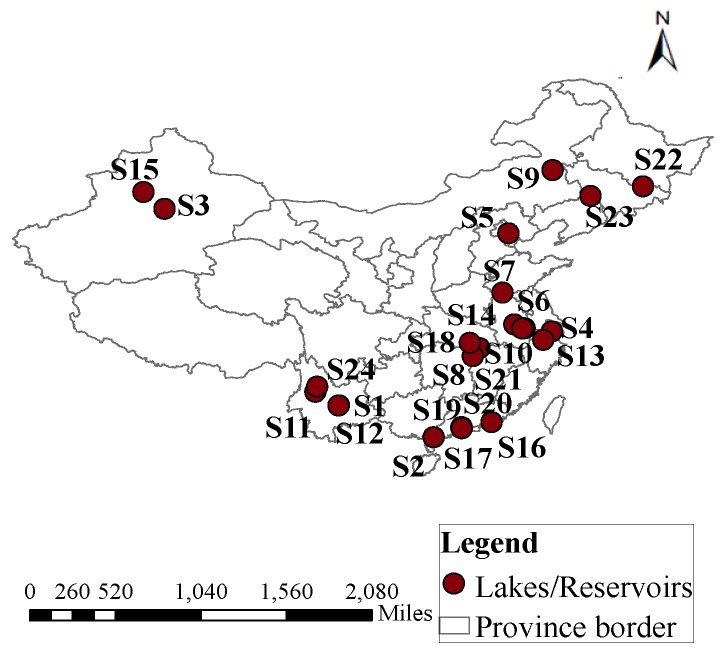
Locations of 24 typical lakes/reservoirs in China.

In this study, eutrophication evaluations of 24 typical lakes/reservoirs in China were performed. The locations of 24 lakes/reservoirs are shown in Figure 1. According to the eutrophication features of lakes/reservoirs, ChlorophyII-a (Chl-a), chemical oxygen demand (COD_Mn_), total phosphorus (TP), total nitrogen (TN), and Secchi Disk (SD) were selected as key indicators for eutrophication evaluation. The data for Chl-a, COD_Mn_, TP, TN, and SD, were monitored by branches of the Ministry of Environmental Protection and the Ministry of Water, and annual mean values are shown in Table 1 [22]. In the State Environmental Protection Administration (SEPA), the water quality standards of level III for Chl-a, COD_Mn_, TP, TN, and SD were regulated as 10.00 mg/m^3^, 8.00 mg/L, 25 mg/m^3^, 300 mg/m^3^, and 2.50 m, respectively.

### 2.2. Cloud-Matter Element (CME) Model

Cloud-matter element is generated by combination of matter element model and cloud model. Matter element (ME) is defined as an ordered triple “object, characteristic, and value” in terms of *R* = (*N*, *c*, *v*), which means that object *N* has *m* characteristic eigenvectors $\left\{c_{1},c_{2},\ldots ,c_{m}\right\}$ with *m* values for eigenvectors $\left\{v_{1},v_{2},\ldots ,v_{m}\right\}$, expressed by Equation (1) as follows [5].

(1) $$R=(N,C,V)=\left[\begin{array}{c} R_{1}\\ R_{2}\\ \ldots \\ R_{m} \end{array}\right]=\left[\begin{array}{ccc} N & c_{1} & v_{1}\\ & c_{2} & v_{2}\\ & \ldots & \ldots \\ & c_{m} & v_{m} \end{array}\right]$$

where $R_{i}=(N,c_{i},v_{i})$, *i* = 1, 2, …, *m*, is called as sub-ME of *m*-dimension ME. 

Suppose each eigenvector has *n* classifications, the *m*-dimension ME for the *k*th evaluated object can be expressed by Equation (2) as follows [14].

(2) $$R_{mn}^{k}=\left[\begin{array}{ccccc} & G_{1} & G_{2} & \ldots & G_{n}\\ C_{1} & x_{11}^{k} & x_{12}^{k} & \ldots & x_{13}^{k}\\ C_{2} & x_{21}^{k} & x_{22}^{k} & \ldots & x_{23}^{k}\\ \ldots & \ldots & \ldots & \ldots & \ldots \\ C_{m} & x_{m1}^{k} & x_{m2}^{k} & \ldots & x_{mn}^{k} \end{array}\right]$$

where $R_{mn}^{k}$ is the ME matrix of the *k*th evaluated object, $x_{ij}^{k}$ is the eigenvector value of the *i*th indicator to the *j*th classification for the *k*th evaluated object, *C_i_* is the *i*th eigenvector, *i* = 1, 2, …, *m*, *G_j_* is the *j*th classification, *j* = 1, 2, …, *n*.

Based on fuzzy set theory and probability theory, cloud model incorporates fuzziness and randomness represented by membership function and probabilistic distribution [23]. Various cloud models such as normal, triangular, trapezoidal, symmetric cloud models were widely applied in the fields of information sciences, such as data mining [24], image segmentation [25], spatial clustering [26], risk assessment [27], uncertainty reasoning [28], and time series prediction [23]. Among them, normal cloud model is the most popular cloud model, which are expressed by three descriptors including expectation Ex, the entropy En, and the hyper-entropy He [28,29]. In this study, the normal cloud model is selected for constructing the CME model. By introducing the cloud model, elements in the ME model can be replaced by certainty degree.

Suppose *U* is the universe of discourse, and *T* is a qualitative concept in *U*. Given x(x∈U) is a random instantiation of concept *T*, and *x* can satisfy $x\sim N(E_{x},E_{n}^{\prime 2})$ and $E_{n}^{\prime }\sim N(E_{n},H_{e}^{2})$, then μ∈[0,1] is the certainty degree of *x* in the universe *U* expressed by Equation (3) as follows [29].

(3) $$\mu =\exp[-\frac{1}{2}\frac{{\left(x-E_{x}\right)}^{2}}{{\left(E_{n}^{\prime }\right)}^{2}}]$$

The distribution of *x* in the universe *U* is called a normal cloud (simplified as cloud in the following sections of the paper), and each *x* with the certainty degree μ is defined as a cloud drop, expressed as (x,μ). The three parameters *E_x_*, *E_n_*, and *H_e_* were determined by Equations (4)–(6) as follows:

(4) $$E_{x}=(S_{\min}+S_{\max})/2,$$

(5) $$E_{n}=(S_{\max}-S_{\min})/6,$$

(6) $$H_{e}=k\cdot E_{n},$$

where *S_min_* and *S_max_* represent the minimum and maximum values of the particular variable. Here, *k* is assumed as 0.1 to balance the variation and robustness of assessment.

### 2.3. Eutrophication Assessment with CME Model

In the eutrophication evaluation, the CME model expressed by Equation (2) is composed of *m* eutrophication evaluation indicators corresponding to *n* evaluation levels for the *k*th lake/reservoir, $x_{ij}^{k}$ is the membership degree of the *i*th indicator to the *j*th level for the *k*th lake/reservoir, which can be obtained through cloud model.

**Figure 2:**
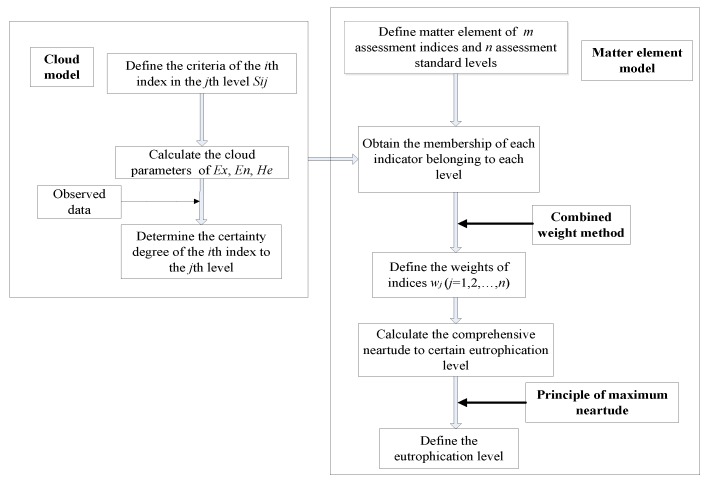
Flowchart of the cloud matter element (CME) model.

The flowchart of the CME model is shown in Figure 2.

The process of the CME-based eutrophication evaluation is expressed as follows:

1. Determine the *m* eutrophication evaluation indicators and *n* classification levels, and the scopes of each evaluation indicator classified as certain eutrophication level.

Based on Chinese environmental legislation, eutrophication is classified into six levels, specified in Table 2 [22].

2. Calculate parameter groups $\left(E_{xij},E_{nij},H_{eij}\right)$ (*i* = 1, 2, …, *m*, *j =* 1, 2, …, *n*) of cloud model by Equations (4)–(6), and shown in Table 3, which are in accordance with the eutrophication classification of five indicators presented in Table 2. The parameters *S*_min_ and *S*_max_ are boundary values of the indicators corresponding to a certain eutrophication level, obtained from Table 2. Since the upper boundaries of Chl-a, COD_Mn_, TP, and TN for level VI, as well as upper boundary of SD for level I are not available, non-linear regression analysis was performed with the assumption of upper boundary values increases with the level. The results obtained for upper boundary were 255.36 mg/m^3^, 28.71 mg/L, 731.07 mg/m^3^, 7770 mg/m^3^, and 18.68 m for Chl-a, COD_Mn_, TP, TN, and SD, respectively.
3. Establish CME matrix with consideration of *m* evaluation factors belonging to certain eutrophication level. Generate the normal random number $E_{n}^{\prime }$ with the expectation value of $E_{n}$ and standard deviation of $H_{e}$. Therefore, the CME model can be expressed by Equation (7) as follows:
  (7) $${\bar{R}}_{mn}^{k}=\left[\begin{array}{ccccc} & G_{1} & G_{2} & \ldots & G_{n}\\ C_{1} & r_{11}^{k} & r_{12}^{k} & \ldots & r_{13}^{k}\\ C_{2} & r_{21}^{k} & r_{22}^{k} & \ldots & r_{23}^{k}\\ \ldots & \ldots & \ldots & \ldots & \ldots \\ C_{m} & r_{m1}^{k} & r_{m2}^{k} & \ldots & r_{mn}^{k} \end{array}\right]=\left[\begin{array}{ccccc} & G_{1} & G_{2} & \ldots & G_{n}\\ C_{1} & {\left(E_{x},E_{n},H_{e}\right)}_{11}^{k} & {\left(E_{x},E_{n},H_{e}\right)}_{12}^{k} & \ldots & {\left(E_{x},E_{n},H_{e}\right)}_{13}^{k}\\ C_{2} & {\left(E_{x},E_{n},H_{e}\right)}_{21}^{k} & {\left(E_{x},E_{n},H_{e}\right)}_{22}^{k} & \ldots & {\left(E_{x},E_{n},H_{e}\right)}_{23}^{k}\\ \ldots & \ldots & \ldots & \ldots & \ldots \\ C_{m} & {\left(E_{x},E_{n},H_{e}\right)}_{m1}^{k} & {\left(E_{x},E_{n},H_{e}\right)}_{m2}^{k} & \ldots & {\left(E_{x},E_{n},H_{e}\right)}_{mn}^{k} \end{array}\right]$$
  where ${\bar{R}}_{mn}^{k}$ is the CME matrix of the *k*th evaluated object and $r_{ij}^{k}$ is the membership degree with distribution parameters ${\left(E_{x},E_{n},H_{e}\right)}_{ij}^{k}$ of the *i*th indicator to the *j*th level for the *k*th evaluated object calculated by Equation (3) [30,31]. Substitute the eigenvector value $x_{ij}^{k}$ into cloud models repeatedly to obtain the distributions of certainty degree and final outcomes corresponding to all classifications.
4. Repeat above steps *N* times to get *N* certainty degrees in CME model.
5. Calculate weights of indicators by combined weight method.

The weights of indicators were determined by means of combined weighing method. Since limitations existed in both entropy method and analytic hierarchy process (AHP) method, the combination of two methods can reflect the influence of the subjective and objective factors, take advantages of both entropy method and AHP method, and avoid defects of the two methods. The combined weights of indicators based on entropy method and AHP method were expressed by Equation (8) as follows:

(8) $$w_{j}=(1-\alpha )w_{j}^{\prime }+\alpha w_{j}^{*}$$

where $w_{j}$ refers to the combined weight of indicators, $w_{j}^{\prime }$ is the weight determined by AHP method, $w_{j}^{*}$ is the weight determined by entropy method, and α is proportion of entropy weight in combined weight calculated by Equation (9) as follows [32].

(9) $$\alpha =\frac{n}{n-1}[(\frac{2}{n}(w_{1}^{\prime }+2w_{2}^{\prime }+\ldots +{nw}_{n}^{\prime })-\frac{n+1}{n})]$$

where $w_{1}^{\prime }$, $w_{2}^{\prime }$, …, $w_{n}^{\prime }$ are AHP-based weights ranged from small to large, and *n* is the numbers of indicators.

The weights of indicators were calculated by Equations (8) and (9) and are shown in Table 4.

6. Calculate neartude of evaluated lakes/reservoirs to certain eutrophication level.

Determine the eutrophication level of evaluated lakes/reservoirs by the principle of maximum neartude.

The neartude measures the proximity between evaluated and standard samples. The application of neartude method avoids negative values of correlative degree method and outperforms the traditional method matter element theory [33]. The greater the neartude to a certain eutrophication level, the more probable it is that the samples belong to the eutrophication level [14]. The neartude of the *k*th evaluated object to the *j*th eutrophication level $\rho H_{j}^{k}$ is represented by Hamming neartude (*ρ*H), which is expressed by Equation (10) as follows:

(10) $$\rho H_{j}^{k}=1-\sum_{j=1}^{n}w_{i}\left|r_{ij}^{k}-r_{i0}\right|$$

where $w_{i}$ is the weights of indicators, *i* = 1, 2, …, *m* and $r_{i0}$ is the element of the ideal normalized matter element matrix, expressed by Equation (11) as follows:

(11) $$R_{i0}=\left|\begin{array}{c} r_{10}\\ r_{20}\\ \ldots \\ r_{m0} \end{array}\right|=\left|\begin{array}{c} 1\\ 1\\ \ldots \\ 1 \end{array}\right|.$$

The definition of eutrophication level is decided by principle of maximum neartude. Supposing $\rho H_{j}^{k}=\max\{\rho H_{j}^{k}\}$, (*j* = 1, 2, ..., *n*), then the lake/reservoir to be evaluated belongs to the *j*th level.

The evaluation process was performed by crystal ball software, which is applied as an analytical tool to help execute, analyze, and make decisions by performing simulations and forecast of data on spreadsheet models [34].

## 3. Results and Discussions

### 3.1. Eutrophication Evaluation Results 

The eutrophication levels of 24 lakes/reservoirs were determined by the CME model, shown in Table 5.

### 3.2. Comparison with Other Methods

A comparative analysis was made between the CME model and other common methods (comprehensive index (CI) method, ME method, FME model, and cloud model), shown in Table 6. Most of the results obtained by the CME model are consistent with the results of other methods. In some lakes, the results of CME model are inconsistent with other methods, and the reasons were analyzed and described as follows.

(1) The results of CME model were consistent with other methods, which verify the validity of the CME model. For example, for Bosten Lake (S3), Ci Lake (S8), Chao Lake (S10), Dianchi Lake (Outer sea) (S11), Dianchi Lake (Cao Sea) (S12), Mogu Lake (S15), Li Lake (S16), Dongshan Lake (S17), Moshui Lake (S18), Liwan Lake (S19), Liuhua Lake (S20), Xuanwu Lake (S21), Jingpo Lake (S22), Nan Lake (S23), and Qionghai Lake (S24), the results of CME model are consistent with the results of all the other evaluation methods. For Yuqiao reservoir (S5), Gucheng Lake (S6), and Nansi Lake (S7), Dali Lake (S9), and West Lake (S13), the results of the CME model are consistent with the results of most of other evaluation methods.
2) In addition, the CME model makes up the limitations of other evaluation methods.
  1) The comprehensive index (CI) method can evaluate eutrophication status comprehensively with consideration of all evaluated indicators. Since the tropical level index (TLI) for COD_Mn_, TP, TN, and SD have close relationships to concentrations of Chl-a, if Chl-a concentration is abnormal by external effects, the evaluation results are inaccurate, which is the limitation of CI method. The results of Dianshan Lake (S4), Yuqiao reservoir (S5), Gucheng Lake (S6), West Lake (S13), and Gantang Lake (S14) obtained by the CI method and the CME model are different. For Dianshan Lake (S4), Yuqiao reservoir (S5), West Lake (S13), and Gantang Lake (S14), the eutrophication levels obtained by the CI method are IV, IV, V, and V, respectively, while the eutrophication levels obtained by the CME model are V, V, VI, and VI, respectively. As such, for most lakes, the eutrophication levels obtained by the CI method are lower than eutrophication levels obtained by the CME model, which proved that the CI method is more conservative than the CME model.
  2) Models of matter element (ME), fuzzy matter element (FME), and cloud matter element (CME) take the eutrophication evaluation process as multi-criteria decision-making process and reflect the impact of all indicators comprehensively and objectively [6].

**Figure 3:**
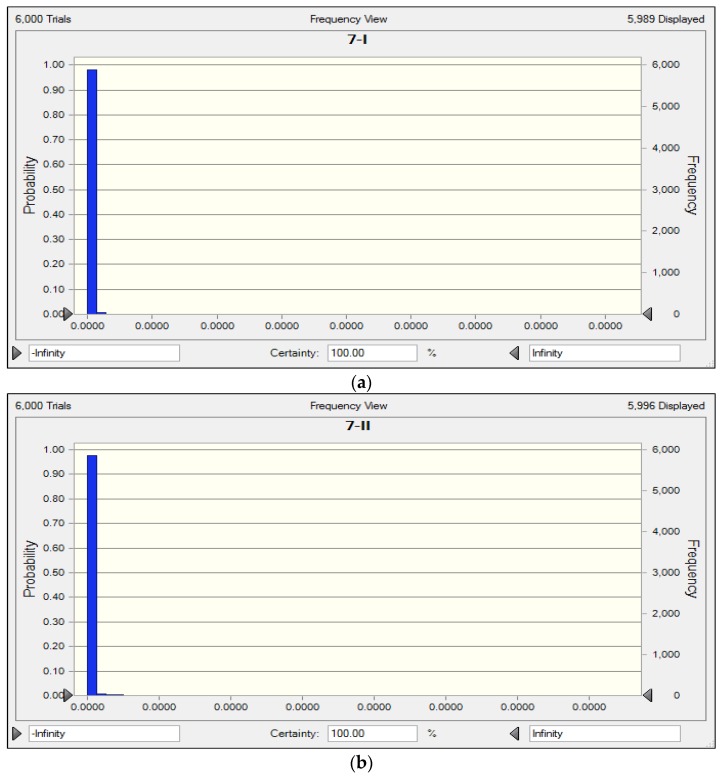

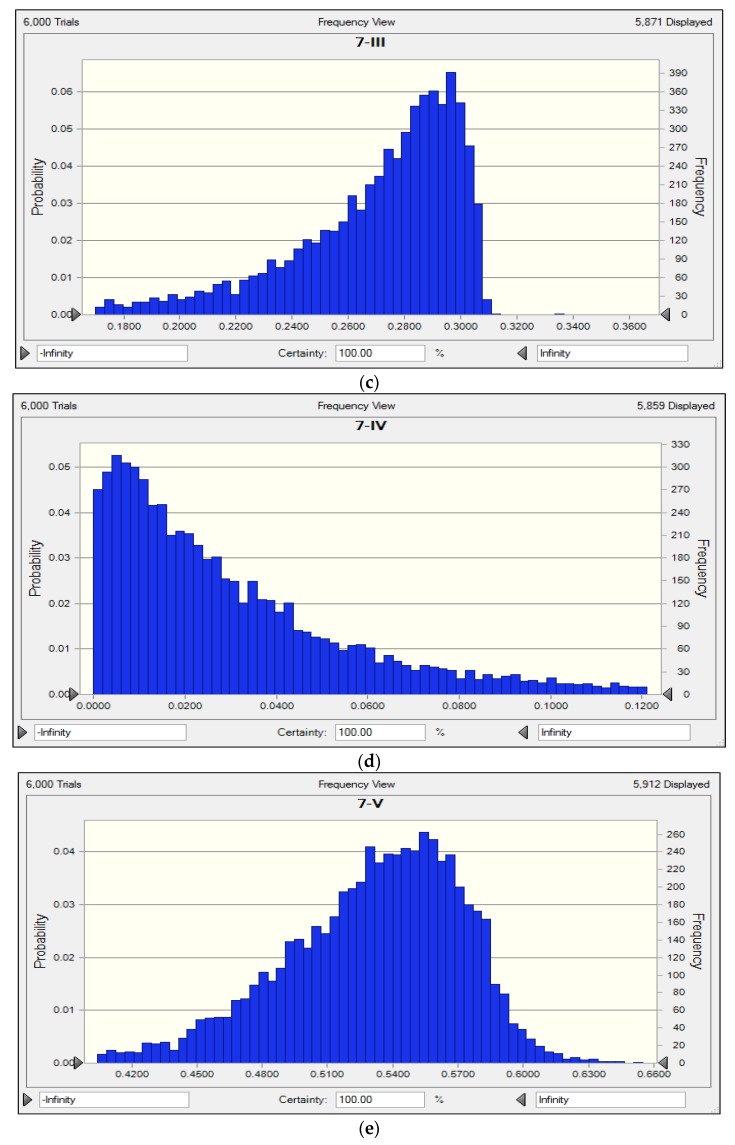

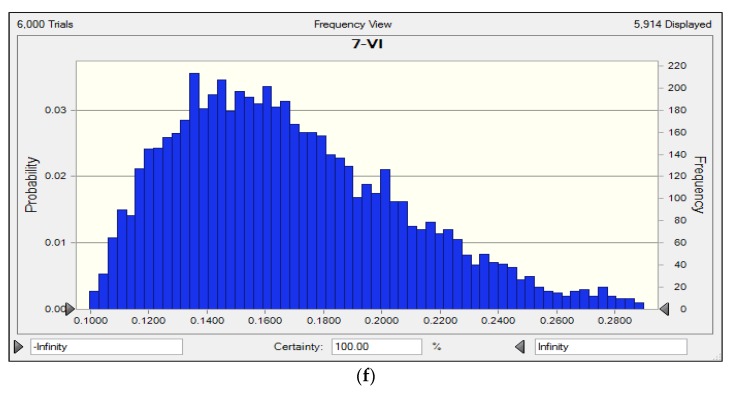
Results of certainty degrees to eutrophication level I, II, III, IV, V, and VI for Nansi Lake. (**a**) Distribution of certainty degree to level I for Nansi Lake (S7); (**b**) Distribution of certainty degree to level II for Nansi Lake (S7); (**c**) Distribution of certainty degree to level III for Nansi Lake (S7); (**d**) Distribution of certainty degree to level IV for Nansi Lake (S7); (**e**) Distribution of certainty degree to level V for Nansi Lake (S7); (**f**) Distribution of certainty degree to level VI for Nansi Lake (S7).

Compared with the ME model, the CME model considers the fuzziness of evaluation criteria and randomness of input data of the model. In the CME model, the certainty degrees of indicators to certain eutrophication degree are not definite, but with certain distribution patterns. For example, the certainty degrees to eutrophication levels for Nansi Lake (S7) are shown in Figure 3. However, in the ME model, the distances between evaluated matter element and standard matter element, the correlation functions, and the complex correlation degrees are distinct without randomness, which makes the evaluation results of the ME model different from results of the CME model for Erhai Lake (S1), Gaoshan Lake (S2), Dianshan Lake (S4), and Dali Lake (S9).

In the fuzzy matter element (FME) model, the fuzziness of eutrophication assessment process is considered. The distances between evaluated matter element and standard matter element are replaced with membership function in fuzzy set theory. The membership functions have multiple patterns including triangular function, trapezoidal function, and normal function. However, the randomness existed in evaluation process was not considered. In the CME model, the membership degrees are replaced by certainty degree with three parameters *E_x_*, *E_n_*, and *H_e_* with consideration of randomness.

**Figure 4:**
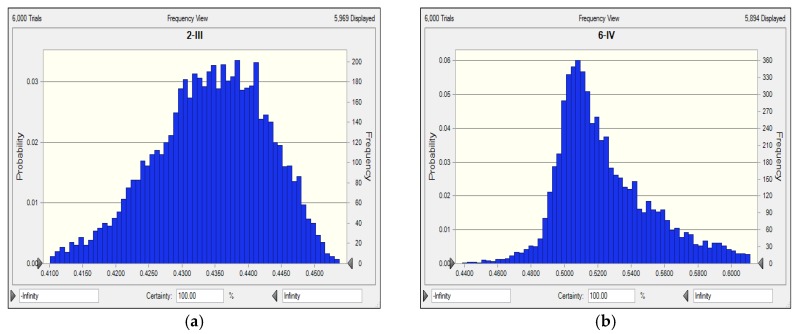

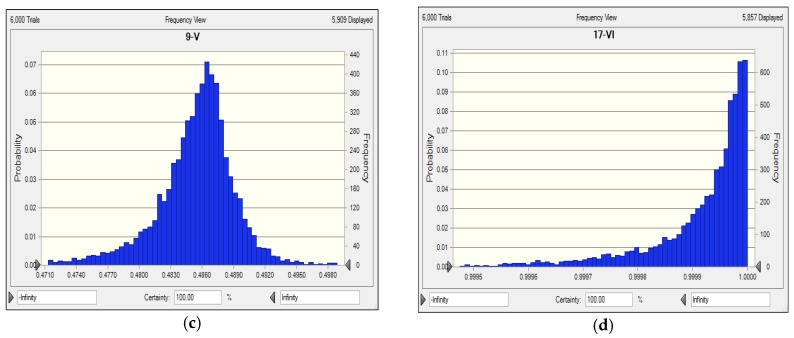
Distribution of certainty degree to level III, IV, V, and VI for (**a**) Distribution of certainty degree to level III for Gaoshan Lake (S2), (**b**) Distribution of certainty degree to level IV for Gucheng Lake (S6), (**c**) Distribution of certainty degree to level V for Dali Lake (S9), and (**d**) Distribution of certainty degree to level VI for Dongshan Lake (S17).

For example, for Gaoshan Lake (S2), Gucheng Lake (S6), Dali Lake (S9), and Dongshan Lake (S17), the eutrophication evaluation results of FME model and CME model were consistent completely, which are level III, IV, V, and VI, respectively. The maximum fuzzy correlation coefficients obtained by the FME model were definite, which were 0.583, 0.477, 0.511, and 0.842 for S2, S6, S9, and S17, respectively. However, the distributions of certainty degree can be obtained by the CME model (shown in Figure 4).

Moreover, the results of the CME model can provide more information than the FME model and ME model. For example, the evaluation results of the CME model and FME model for Dianchi Lake (Cao Sea) (S12) and Liwan Lake (S19) were the same of level VI (shown in Table 7). The comparison between the two lakes cannot be performed by eutrophication results of either the ME model or FME model. However, by the results of the CME model, both the mean value and standard deviation were obtained, and shown in Table 7. The standard deviation reflects the dispersion degree of results from the mean values. It can be found that the eutrophication trend for S12 was more serious than S19 since the standard deviation of S12 (0.0000) was smaller than S19 (0.0016), which means that the results for S12 is more focused to level VI than S19. The comparison of ME, FME, and CME for 24 lakes can be found in Appendix A.

In the cloud model, the fuzziness and randomness in the process of eutrophication evaluation are considered. However, the incompatibilities of indicators are not considered sufficiently. With combination of matter element method in CME model, the incompatible problems are solved efficiently, and more reasonable and objective results can be obtained. For Erhai Lake (S1), Gaoshan Lake (S2), and Nansi Lake (S7), the evaluation results of CME model are different from cloud model. The comparison of certainty degree and neartude were listed in Table 8. The comparison of cloud model and CME for 24 lakes can be found in Appendix B.

The CME model is based on the cloud model, which considers the distance between the evaluated cloud matter element and standard matter element. However, the cloud model does not consider the standard matter element, which makes the differences of evaluation results. Compared with cloud model, the CME model is more complex and scientific.

The CME model has advantages of reducing subjective factors in eutrophication evaluation and avoiding inaccurate assessment results in case of obvious attribution of certain indicators [11]. Therefore, it can deal with uncertainty, fuzziness, and randomness scientifically, and it can solve the complex and contradictory eutrophication evaluation.

(3) Through calculation of neartudes, the eutrophication levels of lakes/reservoirs can be determined more directly. For example, the eutrophication indicators are in different eutrophication levels for Yuqiao Reservoir (S5), i.e., eutrophication levels V, IV, III, V, and IV for Chl-a, COD_Mn_, TP, TN, and SD, respectively. Through the neartude calculation of the CME model, the neartudes of S5 corresponding to each eutrophication level are 0.0000, 0.0000, 0.0311, 0.3604, 0.6022, and 0.0064 for I, II, III, IV, V, and VI, respectively. Therefore, according to the principle of maximum neartude, the eutrophication levels of S5 were level V.
(4) The CME model can judge the eutrophication trend of different lakes and reservoirs with the same eutrophication level. Taking Gantang Lake (S14) and Mogu Lake (S15) as examples, the neartudes of S14 were to level V and VI were 0.3760 and 0.6239, while the neartudes of S15 were to level V and VI were 0.2513 and 0.7486. The levels of both lakes were level VI, however, the eutrophication trend of S15 was more obvious than S14, which is similar to other research results [35]. For the other methods, it is difficult to judge eutrophication degree of different waters with the same eutrophication level.

Compared with other methods, the CME model can not only determine the eutrophication level of lakes/reservoirs, but also judge eutrophication degree of lakes/reservoirs with the same eutrophication level, which makes CME model more advantageous than other evaluation methods.

## 4. Conclusions

In this study, a cloud matter element (CME) model is proposed with combination of cloud model and matter element (ME) model and applied to assess the eutrophication levels of 24 typical lakes and reservoirs in China. The CME model interprets the advantages of both ME model and cloud model with consideration of fuzziness and randomness of eutrophication evaluation process. The weights of indicators were determined by combined method. The eutrophication statuses of lakes and reservoirs were determined according to the maximum neartude to each eutrophication level. Therefore, the proposed CME model can not only evaluate eutrophication level, but also compare eutrophication degree of lakes/reservoirs with the same eutrophication level. The results of the CME model were compared with other methods including comprehensive index (CI) method, matter element (ME) model, fuzzy matter element (FME) model, and cloud model, which verified the correctness of the CME model. In addition, the CME model also makes up the limitations of other methods. For example, the comprehensive assessment index method is influenced by concentration of Chl-a greatly, the matter element (ME) model does not consider the fuzziness of eutrophication evaluation process, the fuzzy matter element model does not consider the randomness of eutrophication evaluation, and has limits on decision of parameters, and the cloud model does not consider the incompatibility of indicators. Therefore, the CME model is more comprehensive, objective, and accurate for evaluating eutrophication levels of lakes and reservoirs. Since the eutrophication is a complex, heterogenous, and specific phenomenon, which vary with time and district, the method proposed can only be applied to judge the eutrophication level preliminarily with some representative indicators. The CME model proposed can be applied to evaluate process in other fields with contradictory, uncertain, fuzzy, and random characteristics.

## Figures and Tables

**Table 1:** Eutrophication data (annual mean values) of 24 typical lakes and reservoirs in China.

Cases	Names	Location (Province/City)	Chl-a (mg/m^3^)	COD_Mn_ (mg/L)	TP (mg/m^3^)	TN (mg/m^3^)	SD (m)
S1	Erhai Lake	Yunnan	4.33	3.38	21	180	2.40
S2	Gaozhou Reservoir	Guangdong	1.49	1.47	46	358	1.72
S3	Bosten Lake	Xinjiang	4.91	5.42	50	969	1.46
S4	Dianshan Lake	Shanghai	3.00	2.87	29	1086	0.67
S5	Yuqiao Reservoir	Tianjin	16.20	5.16	26	1020	1.16
S6	Gucheng Lake	Jiangsu	4.99	2.75	52	2374	0.28
S7	Nansi Lake	Shandong	3.77	6.96	194	3201	0.44
S8	Ci Lake	Hubei	15.38	4.4	87	1540	0.65
S9	Dali Lake	Inner Mongolia	7.24	16.25	153	1671	0.48
S10	Chao Lake	Anhui	14.56	4.34	140	2270	0.27
S11	Dianchi Lake (Outer sea)	Yunnan	44.43	7.11	108	1309	0.49
S12	Dianchi Lake (Cao Sea)	Yunnan	298.86	16.58	931	15,273	0.23
S13	West Lake	Zhejiang	95.94	10.18	136	2230	0.37
S14	Gantang Lake	Jiangxi	77.70	6.96	135	2140	0.36
S15	Mogu Lake	Xinjiang	82.40	14.60	332	2660	0.49
S16	Li Lake	Guangdong	119.51	9.92	372	3038	0.34
S17	Dongshan Lake	Guangdong	185.10	14.80	670	7200	0.26
S18	Moshui Lake	Hubei	262.40	13.60	500	16,050	0.15
S19	Liwan Lake	Guangdong	162.92	14.46	743	7337	0.31
S20	Liuhua Lake	Guangdong	323.51	25.26	643	6777	0.15
S21	Xuanwu Lake	Jiangsu	202.10	8.86	708	6790	0.31
S22	Jingpo Lake	Jilin	4.96	5.96	316	1270	0.73
S23	Nan Lake	Jilin	120.60	8.22	228	2630	0.22
S24	Qionghai Lake	Sichuan	0.88	1.43	130	410	2.98

**Table 2:** Criteria of grading index for eutrophication of lake.

Rank	Chl-a (mg/m^3^)	COD_Mn_ (mg/L)	TP (mg/m^3^)	TN (mg/m^3^)	SD (m)
I	≤0.5	≤0.15	≤1	≤20	≥10
II	≤1	≤0.4	≤4	≤50	≥5
III	≤4	≤2.0	≤25	≤300	≥1.5
IV	≤10	≤4.0	≤50	≤500	≥1.0
V	≤64	≤10.0	≤200	≤2000	≥0.4
VI	>64	>10	>200	>2000	<0.4

**Table 3:** Cloud model parameters of eutrophication levels of all criteria.

Rank	Chl-a (mg/m^3^)			COD_Mn_ (mg/L)			TP (mg/m^3^)			TN (mg/m^3^)			SD (m)		
	*E_x_*	*E_n_*	*H_e_*	*E_x_*	*E_n_*	*H_e_*	*E_x_*	*E_n_*	*H_e_*	*E_x_*	*E_n_*	*H_e_*	*E_x_*	*E_n_*	*H_e_*
I	0.50	0.17	0.02	0.08	0.03	0.00	1.25	0.42	0.04	15.00	5.00	0.50	14.34	1.45	0.14
II	1.50	0.17	0.02	0.28	0.07	0.01	3.75	0.42	0.04	40.00	3.33	0.33	7.50	0.83	0.08
III	3.00	0.33	0.03	1.20	0.33	0.03	15.00	3.33	0.33	175.00	41.67	4.17	3.25	0.58	0.06
IV	7.00	1.00	0.10	3.00	0.67	0.07	37.50	4.17	0.42	400.00	33.33	3.33	1.25	0.08	0.01
V	37.50	9.17	0.92	7.00	1.67	0.17	125.00	25.00	2.50	1250.00	250.00	25.00	0.70	0.10	0.01
VI	160.18	31.73	3.17	19.36	4.79	0.48	465.54	88.51	8.85	4885.00	961.67	96.17	0.20	0.07	0.01

**Table 4:** Weights of indicators for eutrophication assessment.

Indicators	Entropy	Entropy Weights	AHP Weights	Combined Weights
Chl-a	3.185	0.151	0.460	0.309
COD_Mn_	4.739	0.259	0.150	0.203
TP	3.826	0.196	0.090	0.142
TN	3.024	0.140	0.050	0.094
SD	4.661	0.254	0.250	0.252

**Table 5:** Results by cloud matter element model-based assessment approach.

Cases	Neartude						Eutrophication Levels
	I	II	III	IV	V	VI	
Erhai Lake (S1)	0.00	0.00	**0.50**	0.45	0.05	0.00	**III**
Gaoshan Lake (S2)	0.00	0.31	**0.43**	0.24	0.02	0.00	**III**
Bosten Lake (S3)	0.00	0.00	0.06	**0.57**	0.37	0.01	**IV**
Dianshan Lake (S4)	0.00	0.00	0.31	0.33	**0.36**	0.00	**V**
Yuqiao reservoir (S5)	0.00	0.00	0.03	0.36	**0.60**	0.01	**V**
Gucheng Lake (S6)	0.00	0.00	0.00	**0.53**	0.13	0.35	**IV**
Nansi Lake (S7)	0.00	0.00	0.27	0.03	**0.53**	0.17	**V**
Ci Lake (S8)	0.00	0.00	0.00	0.06	**0.94**	0.01	**V**
Dali Lake (S9)	0.00	0.00	0.00	0.31	**0.49**	0.21	**V**
Chao Lake (S10)	0.00	0.00	0.00	0.07	**0.59**	0.35	**V**
Dianchi Lake (Outer sea) (S11)	0.00	0.00	0.00	0.00	**0.99**	0.01	**V**
Dianchi Lake (Cao Sea) (S12)	0.00	0.00	0.00	0.00	0.00	**1.00**	**VI**
West Lake (S13)	0.00	0.00	0.00	0.00	0.29	**0.71**	**VI**
Gantang Lake (S14)	0.00	0.00	0.00	0.00	0.38	**0.62**	**VI**
Mogu Lake (S15)	0.00	0.00	0.00	0.00	0.25	**0.75**	**VI**
Li Lake (S16)	0.00	0.00	0.00	0.00	0.13	**0.87**	**VI**
Dongshan Lake (S17)	0.00	0.00	0.00	0.00	0.00	**0.99**	**VI**
Moshui Lake (S18)	0.00	0.00	0.00	0.00	0.00	**0.99**	**VI**
Liwan Lake (S19)	0.00	0.00	0.00	0.00	0.00	**0.99**	**VI**
Liuhua Lake (S20)	0.00	0.00	0.00	0.00	0.00	**1.00**	**VI**
Xuanwu Lake (S21)	0.00	0.00	0.00	0.00	0.17	**0.83**	**VI**
Jingpo Lake (S22)	0.00	0.00	0.00	0.30	**0.55**	0.15	**V**
Nan Lake (S23)	0.00	0.00	0.00	0.00	0.19	**0.81**	**VI**
Qionghai Lake (S24)	0.30	0.01	**0.44**	0.11	0.15	0.01	**III**

Note: numbers in bold refer to the maximum neartudes.

**Table 6:** Comparison of eutrophication level through various methods.

Cases	Relevant Weighted Nutrition State Comprehensive Index Method						Matter Element (ME) Model	Fuzzy Matter Element (FME) Model	Cloud Model	Cloud Matter Element (CME) Model
	Chl-a	COD_Mn_	TP	TN	SD	Comprehensive Index (CI) Method				
Erhai Lake (S1)	IV	III	III	III	III	III	IV	III	IV	III
Gaoshan Lake (S2)	III	II	IV	III	IV	III	IV	III	IV	III
Bosten Lake (S3)	IV	IV	IV	V	IV	IV	IV	IV	IV	IV
Dianshan Lake (S4)	III	III	III	V	V	IV	IV	V	V	V
Yuqiao reservoir (S5)	V	IV	III	V	IV	IV	V	V	V	V
Gucheng Lake (S6)	IV	III	IV	V	VI	V	IV	IV	IV	IV
Nansi Lake (S7)	III	V	V	VI	V	V	V	V	VI	V
Ci Lake (S8)	V	IV	V	V	V	V	V	V	V	V
Dali Lake (S9)	IV	VI	V	V	V	V	VI	V	V	V
Chao Lake (S10)	V	IV	V	V	VI	V	V	V	V	V
Dianchi Lake (Outer sea) (S11)	V	V	V	V	V	V	V	V	V	V
Dianchi Lake (Cao Sea) (S12)	VI	VI	VI	VI	VI	VI	VI	VI	VI	VI
West Lake (S13)	VI	V	V	V	VI	V	VI	VI	VI	VI
Gantang Lake (S14)	VI	V	V	V	VI	V	V	VI	VI	VI
Mogu Lake (S15)	VI	VI	VI	VI	V	VI	VI	VI	VI	VI
Li Lake (S16)	VI	V	VI	VI	VI	VI	VI	VI	VI	VI
Dongshan Lake (S17)	VI	VI	VI	VI	VI	VI	VI	VI	VI	VI
Moshui Lake (S18)	VI	VI	VI	VI	VI	VI	VI	VI	VI	VI
Liwan Lake (S19)	VI	VI	VI	VI	VI	VI	VI	VI	VI	VI
Liuhua Lake (S20)	VI	VI	VI	VI	VI	VI	VI	VI	VI	VI
Xuanwu Lake (S21)	VI	V	VI	VI	VI	VI	VI	VI	VI	VI
Jingpo Lake (S22)	IV	IV	VI	V	V	V	V	V	V	V
Nan Lake (S23)	VI	V	VI	VI	VI	VI	VI	VI	VI	VI
Qionghai Lake (S24)	III	II	V	III	III	III	III	III	III	III

**Table 7:** Membership functions of three assessment methods of matter element (ME) model, fuzzy matter element model (FME) model, and cloud matter element (CME) model for Dianchi Lake (Cao Sea) (S12) and Liwan Lake (S19).

Evaluated Objects	Methods		Certainty Degree/Neartude to Levels					
			I	II	III	IV	V	VI
Dianchi Lake (Cao Sea) (S12)	ME model		−0.88	−0.88	−0.88	−0.86	−0.81	−0.52
	FME model		0.00	0.00	0.03	0.00	0.05	0.81
	CME model	Mean	0.00	0.00	0.00	0.00	0.00	1.00
		S.D.	0.00	0.00	0.00	0.00	0.00	0.0000
Liwan Lake (S19)	ME model		−0.67	−0.66	−0.65	−0.62	−0.52	0.11
	FME model		0.00	0.00	0.04	0.00	0.08	0.80
	CME model	Mean	0.00	0.00	0.00	0.00	0.00	0.99
		S.D.	0.00	0.00	0.00	0.00	0.00	0.0016

Note: S.D. refer to standard deviation.

**Table 8:** Membership functions of cloud model and cloud matter element (CME) model for Erhai Lake (S1), Gaoshan Lake (S2), and Nansi Lake (S7).

Evaluated Objects	Methods		Certainty Degree/Neartude to Levels						Eutrophication Level
			I	II	III	IV	V	VI	
Erhai Lake (S1)	Cloud model		**0.18**	0.25	0.79	**0.86**	0.59	0.49	IV
	CME model	Mean	0.00	0.00	**0.50**	0.45	0.05	0.00	III
		S. D.	0.00	0.00	0.02	0.04	0.04	0.00	
Gaoshan Lake (S2)	Cloud model		0.21	0.50	0.77	**0.87**	0.65	0.48	IV
	CME model	Mean	0.00	0.31	**0.43**	0.24	0.01	0.00	III
		S. D.	0.00	0.00	0.01	0.01	0.01	0.00	
Nansi Lake (S7)	Cloud model		0.16	0.26	0.47	0.57	0.68	**0.69**	VI
	CME model	Mean	0.00	0.00	0.27	0.03	**0.53**	0.17	V
		S. D.	0.00	0.00	0.03	0.03	0.04	0.04	

## Appendix A

Membership functions of three assessment methods of the matter element (ME) model, fuzzy matter element model (FME) model, and cloud matter element (CME) model for 24 Lakes.

**Table A1:** The comparison of ME, FME, and CME for 24 lakes.

Evaluated Objects	Methods		Certainty Degree/Neartude to Levels					
			I	II	III	IV	V	VI
Dianchi Lake (Cao Sea) (S1)	ME model		−0.58	−0.53	−0.14	0.19	−0.42	−0.73
	FME model		0.00	0.01	0.53	0.41	0.25	0.22
	CME model	Mean	0.00	0.00	0.50	0.45	0.05	0.00
		S.D.	0.00	0.00	0.02	0.04	0.04	0.00
Liwan Lake (S2)	ME model		−0.57	−0.49	−0.10	−0.16	−0.46	−0.74
	FME model		0.00	0.01	0.58	0.28	0.21	0.21
	CME model	Mean	0.00	0.31	0.43	0.24	0.01	0.00
		S.D.	0.00	0.00	0.01	0.01	0.01	0.00
Liwan Lake (S3)	ME model		−0.60	−0.57	−0.40	−0.03	−0.06	−0.60
	FME model		0.00	0.00	0.17	0.45	0.44	0.25
	CME model	Mean	0.00	0.00	0.04	0.06	0.04	0.00
		S.D.	0.00	0.00	0.00	0.00	0.00	0.00
Liwan Lake (S4)	ME model		−0.60	−0.57	−0.23	−0.15	−0.39	−0.49
	FME model		0.00	0.01	0.39	0.40	0.54	0.26
	CME model	Mean	0.00	0.00	0.31	0.33	0.36	0.00
		S.D.	0.00	0.00	0.00	0.01	0.01	0.00
Liwan Lake (S5)	ME model		−0.61	−0.58	−0.45	−0.21	0.12	−0.53
	FME model		0.00	0.00	0.16	0.32	0.54	0.25
	CME model	Mean	0.00	0.00	0.03	0.36	0.60	0.01
		S.D.	0.00	0.00	0.03	0.03	0.01	0.00
Liwan Lake (S6)	ME model		−0.61	−0.59	−0.43	−0.15	−0.44	−0.58
	FME model		0.00	0.00	0.10	0.48	0.33	0.42
	CME model	Mean	0.00	0.00	0.00	0.53	0.13	0.35
		S.D.	0.00	0.00	0.00	0.03	0.03	0.00
Liwan Lake (S7)	ME model		−0.61	−0.59	−0.41	−0.38	−0.28	−0.32
	FME model		0.00	0.00	0.23	0.14	0.54	0.41
	CME model	Mean	0.00	0.00	0.27	0.03	0.53	0.17
		S.D.	0.00	0.00	0.03	0.03	0.04	0.04
Liwan Lake (S8)	ME model		−0.61	−0.60	−0.53	−0.36	0.01	−0.20
	FME model		0.00	0.00	0.05	0.06	0.77	0.30
	CME model	Mean	0.00	0.00	0.00	0.06	0.94	0.01
		S.D.	0.00	0.00	0.00	0.02	0.02	0.00
Liwan Lake (S9)	ME model		−0.65	−0.62	−0.55	−0.24	−0.24	−0.22
	FME model		0.00	0.00	0.04	0.31	0.51	0.44
	CME model	Mean	0.00	0.00	0.00	0.31	0.49	0.21
		S.D.	0.00	0.00	0.00	0.00	0.00	0.00
Liwan Lake (S10)	ME model		−0.62	−0.61	−0.55	−0.41	−0.14	−0.56
	FME model		0.00	0.00	0.03	0.06	0.53	0.46
	CME model	Mean	0.00	0.00	0.00	0.07	0.59	0.35
		S.D.	0.00	0.00	0.00	0.02	0.02	0.01
Liwan Lake (S11)	ME model		−0.62	−0.61	−0.57	−0.47	0.16	−0.21
	FME model		0.00	0.00	0.04	0.00	0.91	0.40
	CME model	Mean	0.00	0.00	0.00	0.00	0.99	0.01
		S.D.	0.00	0.00	0.00	0.00	0.01	0.01
Liwan Lake (S12)	ME model		−0.88	−0.88	−0.88	−0.86	−0.81	−0.52
	FME model		0.00	0.00	0.03	0.00	0.05	0.81
	CME model	Mean	0.00	0.00	0.00	0.00	0.00	1.00
		S.D.	0.00	0.00	0.00	0.00	0.00	0.00
Liwan Lake (S13)	ME model		−0.62	−0.62	−0.59	−0.53	−0.32	0.10
	FME model		0.00	0.00	0.04	0.00	0.38	0.54
	CME model	Mean	0.00	0.00	0.00	0.00	0.29	0.71
		S.D.	0.00	0.00	0.00	0.00	0.04	0.04
Liwan Lake (S14)	ME model		−0.59	−0.57	−0.51	−0.38	0.06	−0.08
	FME model		0.00	0.00	0.04	0.00	0.55	0.61
	CME model	Mean	0.00	0.00	0.00	0.00	0.38	0.62
		S.D.	0.00	0.00	0.00	0.00	0.03	0.03
Liwan Lake (S15)	ME model		−0.62	−0.62	−0.59	−0.53	−0.34	0.22
	FME model		0.00	0.00	0.04	0.00	0.23	0.55
	CME model	Mean	0.00	0.00	0.00	0.00	0.25	0.75
		S.D.	0.00	0.00	0.00	0.00	0.00	0.00
Liwan Lake (S16)	ME model		−0.62	−0.60	−0.58	−0.53	−0.36	0.09
	FME model		0.00	0.00	0.04	0.00	0.20	0.63
	CME model	Mean	0.00	0.00	0.00	0.00	0.13	0.87
		S.D.	0.00	0.00	0.00	0.00	0.03	0.03
Liwan Lake (S17)	ME model		−0.68	−0.68	−0.67	−0.64	−0.55	0.05
	FME model		0.00	0.00	0.03	0.00	0.06	0.84
	CME model	Mean	0.00	0.00	0.00	0.00	0.00	1.00
		S.D.	0.00	0.00	0.00	0.00	0.00	0.00
Liwan Lake (S18)	ME model		−0.77	−0.77	−0.76	−0.75	−0.68	−0.26
	FME model		0.00	0.00	0.03	0.00	0.03	0.81
	CME model	Mean	0.00	0.00	0.00	0.00	0.00	1.00
		S.D.	0.00	0.00	0.00	0.00	0.00	0.00
Liwan Lake (S19)	ME model		−0.67	−0.66	−0.65	−0.62	−0.52	0.11
	FME model		0.00	0.00	0.04	0.00	0.08	0.80
	CME model	Mean	0.00	0.00	0.00	0.00	0.00	0.99
		S.D.	0.00	0.00	0.00	0.00	0.00	0.0016
Liwan Lake (S20)	ME model		−0.88	−0.88	−0.88	−0.88	−0.85	−0.48
	FME model		0.00	0.00	0.03	0.00	0.02	0.94
	CME model	Mean	0.00	0.00	0.00	0.00	0.00	1.00
		S.D.	0.00	0.00	0.00	0.00	0.00	0.00
Liwan Lake (S21)	ME model		−0.70	−0.69	−0.68	−0.63	−0.48	0.01
	FME model		0.00	0.00	0.04	0.00	0.23	0.79
	CME model	Mean	0.00	0.00	0.00	0.00	0.17	0.83
		S.D.	0.00	0.00	0.00	0.00	0.01	0.01
Liwan Lake (S22)	ME model		−0.61	−0.59	−0.46	−0.24	−0.16	−0.26
	FME model		0.00	0.00	0.11	0.24	0.65	0.32
	CME model	Mean	0.00	0.00	0.00	0.30	0.55	0.15
		S.D.	0.00	0.00	0.00	0.01	0.01	0.00
Liwan Lake (S23)	ME model		−0.62	−0.62	−0.60	−0.56	−0.30	−0.11
	FME model		0.00	0.00	0.03	0.00	0.27	0.64
	CME model	Mean	0.00	0.00	0.00	0.00	0.19	0.81
		S.D.	0.00	0.00	0.00	0.00	0.01	0.01
Liwan Lake (S24)	ME model		−0.52	−0.30	−0.15	−0.20	−0.45	−0.71
	FME model		0.04	0.28	0.59	0.15	0.29	0.23
	CME model	Mean	0.30	0.01	0.44	0.11	0.15	0.00
		S.D.	0.02	0.01	0.01	0.01	0.01	0.00

## Appendix B

Membership functions of the cloud model and cloud matter element (CME) model for 24 Lakes.

**Table A2:** The comparison of cloud model and CME for 24 lakes.

Evaluated Objects	Methods		Certainty Degree/Neartude to Levels					
			I	II	III	IV	V	VI
Erhai Lake (S1)	Cloud model		0.18	0.25	0.79	0.86	0.59	0.49
	CME model	Mean	0.00	0.00	0.50	0.45	0.05	0.00
		S. D.	0.00	0.00	0.02	0.04	0.04	0.00
Gaoshan Lake (S2)	Cloud model		0.21	0.50	0.77	0.87	0.65	0.48
	CME model	Mean	0.00	0.31	0.43	0.24	0.01	0.00
		S. D.	0.00	0.00	0.01	0.01	0.01	0.00
Nansi Lake (S3)	Cloud model		0.17	0.21	0.48	0.86	0.71	0.51
	CME model	Mean	0.00	0.00	0.04	0.06	0.04	0.00
		S. D.	0.00	0.00	0.00	0.00	0.00	0.00
Nansi Lake (S4)	Cloud model		0.16	0.35	0.66	0.85	0.86	0.51
	CME model	Mean	0.00	0.00	0.31	0.33	0.36	0.00
		S. D.	0.00	0.00	0.00	0.01	0.01	0.00
Nansi Lake (S5)	Cloud model		0.16	0.18	0.31	0.70	0.82	0.52
	CME model	Mean	0.00	0.00	0.03	0.36	0.60	0.01
		S. D.	0.00	0.00	0.03	0.03	0.01	0.00
Nansi Lake (S6)	Cloud model		0.16	0.18	0.51	0.82	0.81	0.74
	CME model	Mean	0.00	0.00	0.00	0.53	0.13	0.35
		S. D.	0.00	0.00	0.00	0.03	0.03	0.00
Nansi Lake (S7)	Cloud model		0.16	0.26	0.47	0.57	0.68	0.69
	CME model	Mean	0.00	0.00	0.27	0.03	0.53	0.17
		S. D.	0.00	0.00	0.03	0.03	0.04	0.04
Nansi Lake (S8)	Cloud model		0.16	0.17	0.19	0.62	0.92	0.55
	CME model	Mean	0.00	0.00	0.00	0.06	0.94	0.01
		S. D.	0.00	0.00	0.00	0.02	0.02	0.00
Nansi Lake (S9)	Cloud model		0.16	0.16	0.29	0.52	0.69	0.68
	CME model	Mean	0.00	0.00	0.00	0.31	0.49	0.21
		S. D.	0.00	0.00	0.00	0.00	0.00	0.00
Nansi Lake (S10)	Cloud model		0.16	0.16	0.17	0.54	0.86	0.78
	CME model	Mean	0.00	0.00	0.00	0.07	0.59	0.35
		S. D.	0.00	0.00	0.00	0.02	0.02	0.01
Nansi Lake (S11)	Cloud model		0.16	0.16	0.18	0.32	0.86	0.67
	CME model	Mean	0.00	0.00	0.00	0.00	0.99	0.01
		S. D.	0.00	0.00	0.00	0.00	0.01	0.01
Nansi Lake (S12)	Cloud model		0.16	0.16	0.16	0.18	0.20	1.00
	CME model	Mean	0.00	0.00	0.00	0.00	0.00	1.00
		S. D.	0.00	0.00	0.00	0.00	0.00	0.00
Nansi Lake (S13)	Cloud model		0.16	0.16	0.17	0.21	0.54	0.83
	CME model	Mean	0.00	0.00	0.00	0.00	0.29	0.71
		S. D.	0.00	0.00	0.00	0.00	0.04	0.04
Nansi Lake (S14)	Cloud model		0.16	0.16	0.17	0.29	0.70	0.81
	CME model	Mean	0.00	0.00	0.00	0.00	0.38	0.62
		S. D.	0.00	0.00	0.00	0.00	0.03	0.03
Nansi Lake (S15)	Cloud model		0.16	0.16	0.18	0.21	0.48	0.78
	CME model	Mean	0.00	0.00	0.00	0.00	0.25	0.75
		S. D.	0.00	0.00	0.00	0.00	0.00	0.00
Nansi Lake (S16)	Cloud model		0.16	0.16	0.17	0.21	0.32	0.90
	CME model	Mean	0.00	0.00	0.00	0.00	0.13	0.87
		S. D.	0.00	0.00	0.00	0.00	0.03	0.03
Nansi Lake (S17)	Cloud model		0.16	0.16	0.16	0.18	0.21	0.98
	CME model	Mean	0.00	0.00	0.00	0.00	0.00	1.00
		S. D.	0.00	0.00	0.00	0.00	0.00	0.00
Nansi Lake (S18)	Cloud model		0.15	0.16	0.16	0.17	0.19	0.99
	CME model	Mean	0.00	0.00	0.00	0.00	0.00	1.00
		S. D.	0.00	0.00	0.00	0.00	0.00	0.00
Nansi Lake (S19)	Cloud model		0.16	0.16	0.17	0.19	0.22	0.96
	CME model	Mean	0.00	0.00	0.00	0.00	0.00	0.99
		S. D.	0.00	0.00	0.00	0.00	0.00	0.00
Nansi Lake (S20)	Cloud model		0.15	0.16	0.16	0.17	0.18	1.00
	CME model	Mean	0.00	0.00	0.00	0.00	0.00	1.00
		S. D.	0.00	0.00	0.00	0.00	0.00	0.00
Nansi Lake (S21)	Cloud model		0.16	0.16	0.17	0.22	0.25	0.94
	CME model	Mean	0.00	0.00	0.00	0.00	0.17	0.83
		S. D.	0.00	0.00	0.00	0.00	0.01	0.01
Nansi Lake (S22)	Cloud model		0.16	0.19	0.44	0.66	0.73	0.57
	CME model	Mean	0.00	0.00	0.00	0.30	0.55	0.15
		S. D.	0.00	0.00	0.00	0.01	0.01	0.00
Nansi Lake (S23)	Cloud model		0.15	0.16	0.16	0.22	0.42	0.93
	CME model	Mean	0.00	0.00	0.00	0.00	0.19	0.81
		S. D.	0.00	0.00	0.00	0.00	0.01	0.01
Nansi Lake (S24)	Cloud model		0.41	0.49	0.73	0.59	0.59	0.49
	CME model	Mean	0.30	0.01	0.44	0.11	0.15	0.00
		S. D.	0.02	0.01	0.01	0.01	0.01	0.00
